# Supplementary material for: Vascular and Liver Homeostasis in Juvenile Mice Require Endothelial Cyclic AMP-Dependent Protein Kinase A
Source: Int J Mol Sci. 2022 Sep 27;23(19):11419. doi: 10.3390/ijms231911419 (PMC9570023; doi:10.3390/ijms231911419)
Supplement: Supplementary file 1 [file ijms-23-11419-s001.zip › ijms-1894614-supplementary/Supplement/ijms-1894614-supplementary.pdf]

Figure S1: Subcutaneous edema in dnPKA^{iEC} mice.

Video S1: MRI imaging of control and dnPKA^{iEC} littermates demonstrate subcutaneous edema in dnPKA^{iEC} mouse.

Figure S2: Hypervascularization of inguinal fat pad in dnPKA^{iEC} mice.

Figure S3: Cdh5-CreERT2- and Vegfr3-CreERT2-induced recombination in the liver.

Figure S4: Disorganization of the hepatic vasculature in the liver of dnPKA^{iEC} mice.
