# Supplementary figures and images for: Vascular and Liver Homeostasis in Juvenile Mice Require Endothelial Cyclic AMP-Dependent Protein Kinase A
Source: Int J Mol Sci. 2022 Sep 27;23(19):11419. doi: 10.3390/ijms231911419 (PMC9570023; doi:10.3390/ijms231911419)

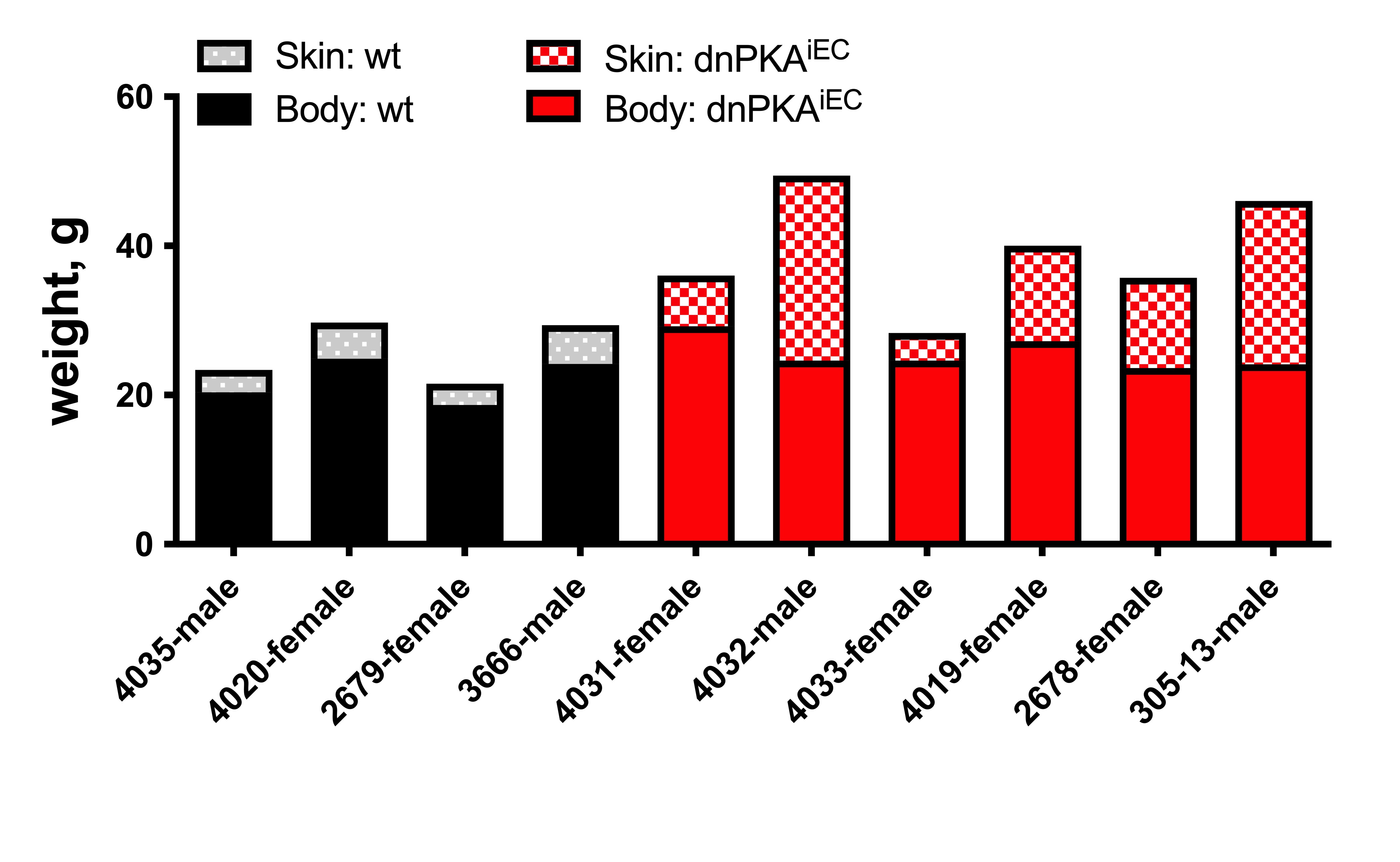

Supplement: Supplementary file 1 [file ijms-23-11419-s001.zip › ijms-1894614-supplementary/Supplement/FigS1.jpg]

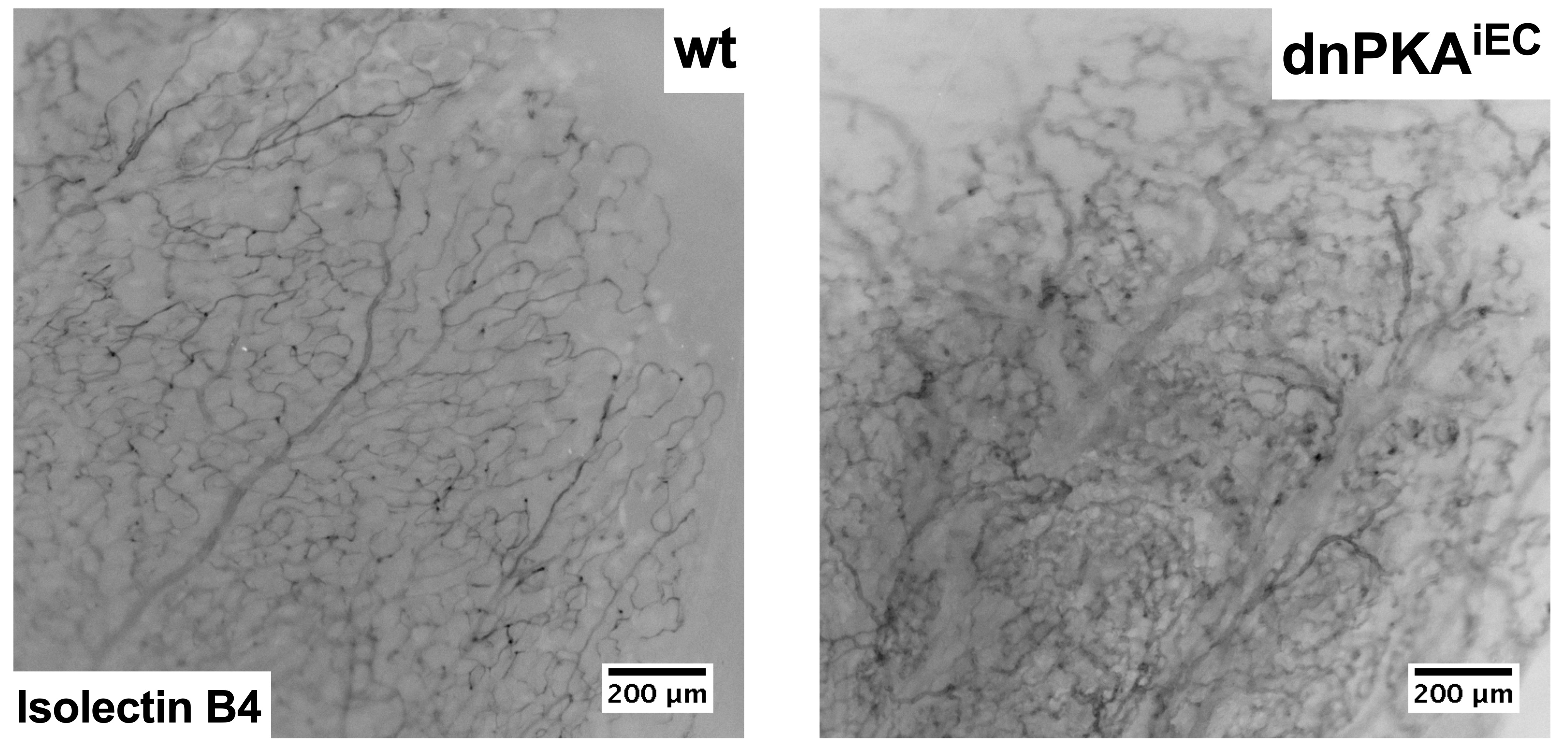

Supplement: Supplementary file 1 [file ijms-23-11419-s001.zip › ijms-1894614-supplementary/Supplement/FigS2.jpg]

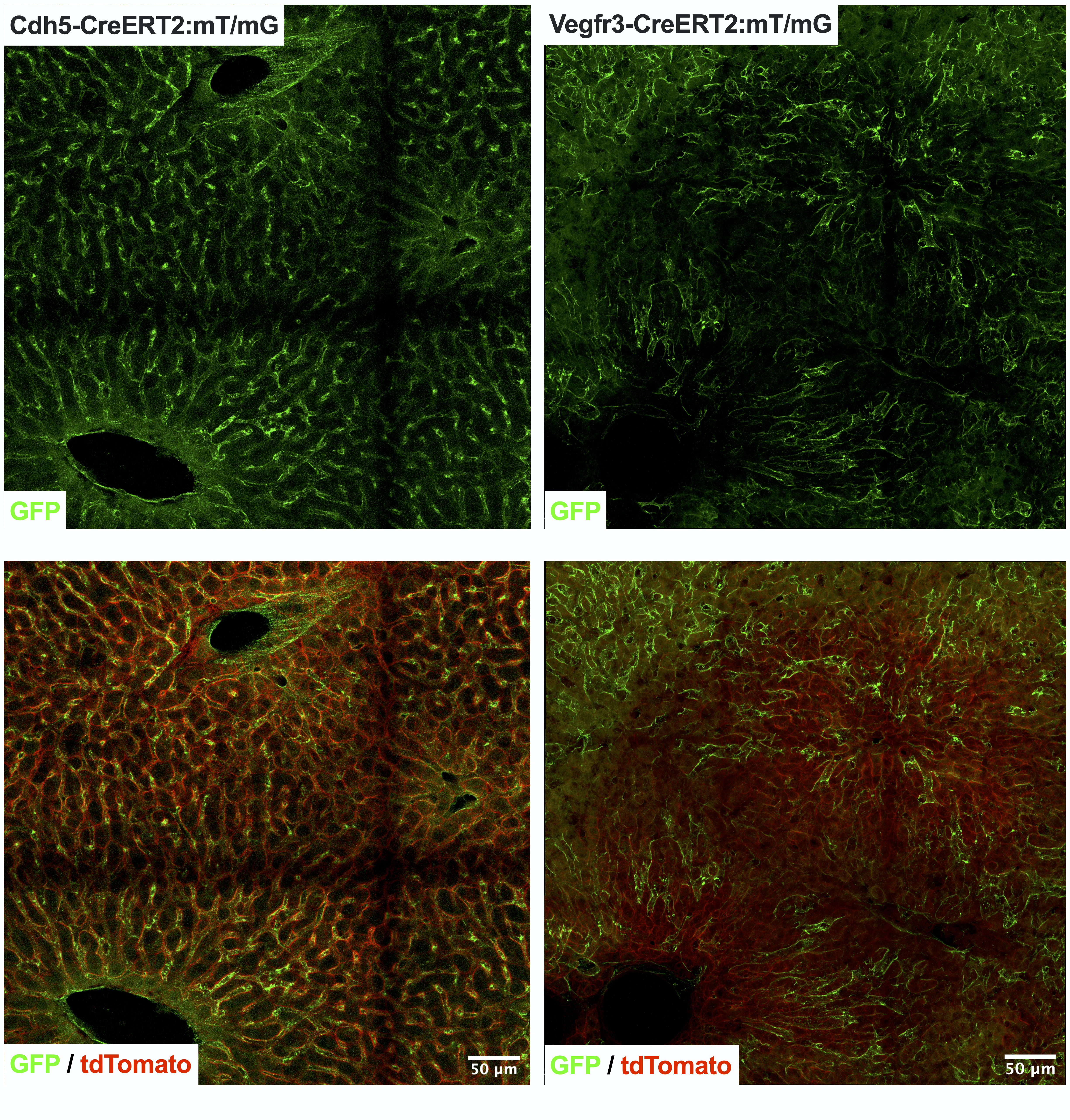

Supplement: Supplementary file 1 [file ijms-23-11419-s001.zip › ijms-1894614-supplementary/Supplement/FigS3_corrected.png]

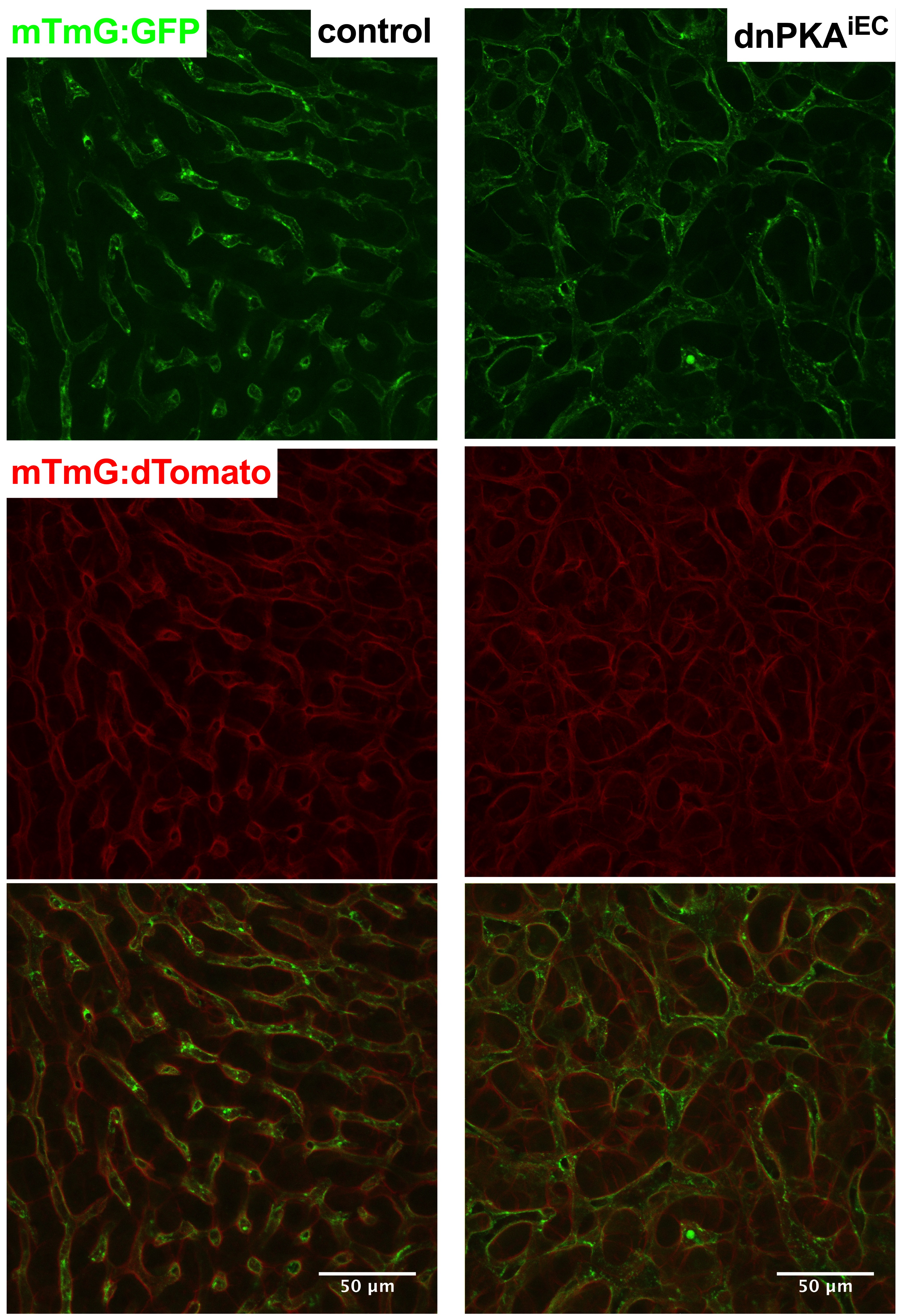

Supplement: Supplementary file 1 [file ijms-23-11419-s001.zip › ijms-1894614-supplementary/Supplement/FigS4.jpg]
